# Syntheses and Structural Investigations of Penta-Coordinated Co(II) Complexes with *Bis*-Pyrazolo-*S*-Triazine Pincer Ligands, and Evaluation of Their Antimicrobial and Antioxidant Activities

**DOI:** 10.3390/molecules26123633

**Published:** 2021-06-14

**Authors:** Saied M. Soliman, Raghdaa A. Massoud, Hessa H. Al-Rasheed, Ayman El-Faham

**Affiliations:** 1 Department of Chemistry, Faculty of Science, Alexandria University, P.O. Box 426, Ibrahimia, Alexandria 21321, Egypt; raghdaamassoud@yahoo.com; 2 Department of Chemistry, College of Science, King Saud University, P.O. Box 2455, Riyadh 11451, Saudi Arabia; halbahli@ksu.edu.sa

**Keywords:** pincer, penta-coordinated Co(II), Hirshfeld, antimicrobial activity, antioxidant activity

## Abstract

Two penta-coordinated **[Co(^Morph^BPT)Cl_2_]**; **1** and **[Co(^Pip^BPT)Cl_2_]**; **2** complexes with the *bis*-pyrazolyl-*s*-triazine pincer ligands **^Morph^BPT** and **^Pip^BPT** were synthesized and characterized. Both **^Morph^BPT** and **^Pip^BPT** act as NNN-tridentate pincer chelates coordinating the Co(II) center with one short Co-N(*s*-triazine) and two longer Co-N(pyrazole) bonds. The coordination number of Co(II) is five in both complexes, and the geometry around Co(II) ion is a distorted square pyramidal in **1,** while **2** shows more distortion. In both complexes, the packing is dominated by Cl…H, C-H…π, and Cl…C (anion-π stacking) interactions in addition to O…H interactions, which are found only in **1**. The UV-Vis spectral band at 564 nm was assigned to metal–ligand charge transfer transitions based on TD-DFT calculations. Complexes **1** and **2** showed higher antimicrobial activity compared to the respective free ligand **^Morph^BPT** and **^Pip^BPT,** which were not active. MIC values indicated that **2** had better activity against *S. aureus, B. subtilis*, and *P. vulgaris* than **1**. DPPH free radical scavenging assay revealed that all the studied compounds showed weak to moderate antioxidant activity where the nature of the substituent at the *s*-triazine core has a significant impact on the antioxidant activity.

## 1. Introduction

Triazine is a prototypal molecule that has, together with its derivatives, a wide commercial uses, for example, in resins, dyes, herbicides, or as sulfide removal agents [1,2]. These compounds are a well-suited model system in molecular imprinting [3]. *s*-Triazines are widely used within the pharmaceutical, textile, plastic, and rubber industries, and as pesticides, dyestuffs, optical bleaches, explosives, and surface-active agents [4,5,6]. *s*-Triazine and its compounds are also used as subunits in the formation of supramolecular structures because they possess good optical and electronic properties and are able to form three strong hydrogen bonds with the host molecule [7].

On the other hand, bacterial and fungal infectious diseases are very common all over the world. Due to the rapid development in drug resistance, tolerance, and side effects, there is a critical need for new antibacterial and antifungal agents that exhibit improved pharmacological properties and drug-resistance profiles [8,9]. In this aspect, the triazine class [10,11,12,13,14,15,16,17,18,19] and their metal complexes [20,21,22,23,24] have been received a great deal of attention as they demonstrate wide range of therapeutic activities and a great array of biological applications including antimicrobial, antituberculosis, anticancer, antiviral, antibacterial, antifungal, anti-HIV, and antimalarial activities [9,25,26]. In addition, a number of metal complexes based on triazine derivatives have been studied for their interesting magnetic [27,28,29] and catalytic [30,31,32,33,34,35,36,37,38,39,40,41] applications.

**Figure 1:**
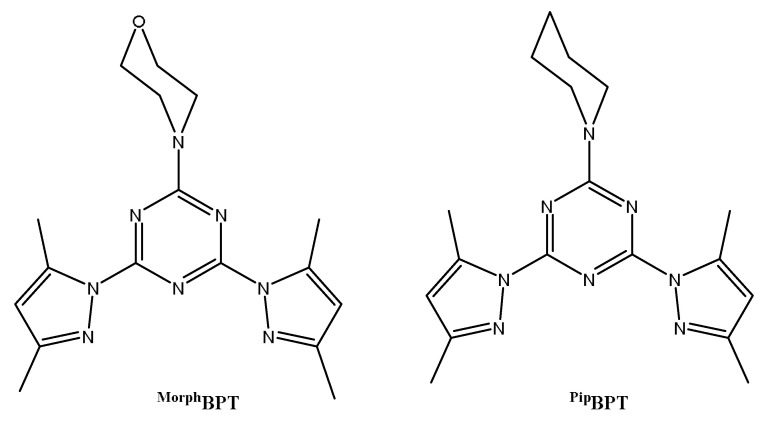
Structure of the pincer ligands.

Among the *s*-triazine derivatives, 2,4-*bis*(3,5-dimethyl-1*H*-pyrazol-1-yl)-6-methoxy-1,3,5-triazine (**MBPT**) pincer ligand has been extensively used in the synthesis of a wide range of homoleptic and heteroleptic metal(II) complexes with coordination numbers ranging from five to eight [42,43,44,45,46,47,48,49,50]. Recently, we reported that the reaction of **MBPT** with CoCl_2_ afforded the [Co(MBPT)Cl(H_2_O)_2_]Cl pincer complex, which was found to be the best candidate as an antimicrobial agent compared to the [Co(MBPT)(NO_3_)_2_] and [Co(MBPT)(H_2_O)_3_](ClO_4_)_2_ analogues [48]. Furthermore, a number of [ML]Cl_2_ and [ML_2_]Cl_2_ complexes (where M = Cu(II), Ni(II), and Co(II), and **L** is 2,4-*bis*(3,5-dimethyl-1*H*-pyrazol-1-yl)-6-phenylamino-1,3,5-triazine) were synthesized and were also investigated for their antimicrobial activities [51]. Following the research on the same class of these pincer ligands, we present here the self-assembly of the *s*-triazine functional ligands shown in Figure 1 with CoCl_2_ in order to synthesize new biologically active complexes bearing both bioactive species; the ligand and Co(II) ion, which could lead to the synthesis of powerful antimicrobial agents. The structural aspects of the synthesized complexes were analyzed using single-crystal X-ray diffraction, Hirshfeld, and DFT calculations, as well as spectroscopic analysis. In addition, the antimicrobial and antioxidant activities of both complexes were examined and compared with the free ligands.

## 2. Results and Discussion

### 2.1. Chemistry

The self-assembly of the functional ligands 4-(4,6-bis(3,5-dimethyl-1*H*-pyrazol-1-yl)-1,3,5-triazin-2-yl)morpholine (**^Morph^BPT**) and 2,4-bis(3,5-dimethyl-1*H*-pyrazol-1-yl)-6-(piperidin-1-yl)-1,3,5-triazine (**^Pip^BPT**) with CoCl_2_ in methanol afforded the corresponding heteroleptic neutral complexes **[Co(^Morph^BPT)Cl_2_] (1)** and **[Co(^pip^BPT)Cl_2_] (2)** in good yield. The Structure aspects of complexes **1** and **2** were analyzed using different spectroscopic tools such as FTIR, UV-Vis, and low-temperature X-ray single-crystal diffraction combined with Hirshfeld calculations. Crystallographic details are listed in Table 1. In addition, biological evaluations of these new complexes as antimicrobial and antioxidant agents were performed and compared with the corresponding free ligands.

#### 2.1.1. Structure Description of **[Co(^Morph^BPT)Cl_2_]** (1)

**Figure 2:**
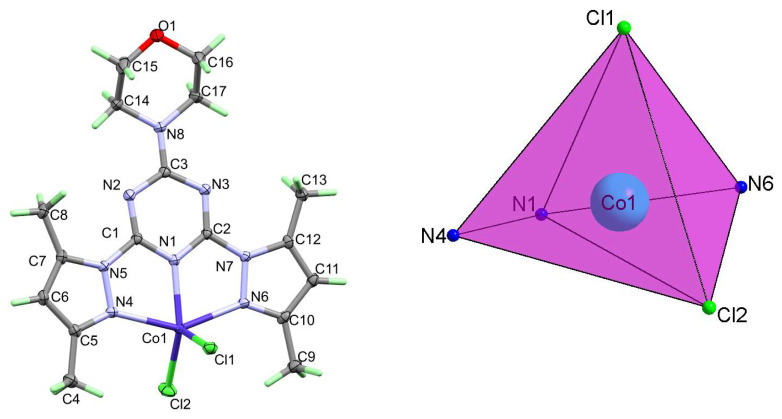
Structure with atom numbering (left) and the distorted square pyramidal (right) of **1**.

The neutral complex **[Co(^Morph^BPT)Cl_2_]** (1) crystallized in the monoclinic crystal system and the centrosymmetric C2/c space group. The asymmetric unit comprised one **[Co(^Morph^BPT)Cl_2_]** with Z = 8. The structure of **1** revealed a penta-coordinated Co(II) complex with one tridentate **^Morph^BPT** chelate and two chloride anionic ligands in the inner sphere (Figure 2; right part). The **^Morph^BPT** ligand coordinated the Co(II) via one short Co-N_(*s*-triazine)_ and relatively longer Co-N_(pyrazole)_ bonds. The corresponding Co1-N1, Co1-N4, and Co1-N6 distances are 2.0387(15), 2.2008(15), and 2.2304(16) Å, respectively. The two Co-Cl distances are very similar, where the Co1-Cl1 and Co1-Cl2 distances are 2.2718(7) and 2.2968(7) Å, respectively. The bite angles in the coordinated **^Morph^BPT** are 74.01(6) and 73.07(5)° for N1-Co1-N4 and N1-Co1-N6, respectively, while the N4-Co1-N6 and Cl2-Co1-Cl1 bond angles are 146.87(5) and 111.40(3)°, respectively (Table 2). The ring systems in **^Morph^BPT** are not perfectly coplanar where the angle between the *s*-triazine mean plane and each of the two pyrazolyl rings are 4.88 and 8.49° for the pyrazole moieties with lower and higher atom numbering, respectively. The distortion in the CoN_3_Cl_2_ coordination sphere of complex **1** was described using the criterion reported by Addison [52]. The geometry around Co(II) is a distorted square pyramidal with N4-Co1-N6 (β= of 146.87(5)°) and N1-Co1-Cl2 (α = 138.89(4)°), giving a τ value of 0.133. The distorted square pyramidal configuration comprised Cl2N1N4N6 donor atoms in the basal plane and Cl1 as apical (Figure 2; left part).

**Figure 3:**
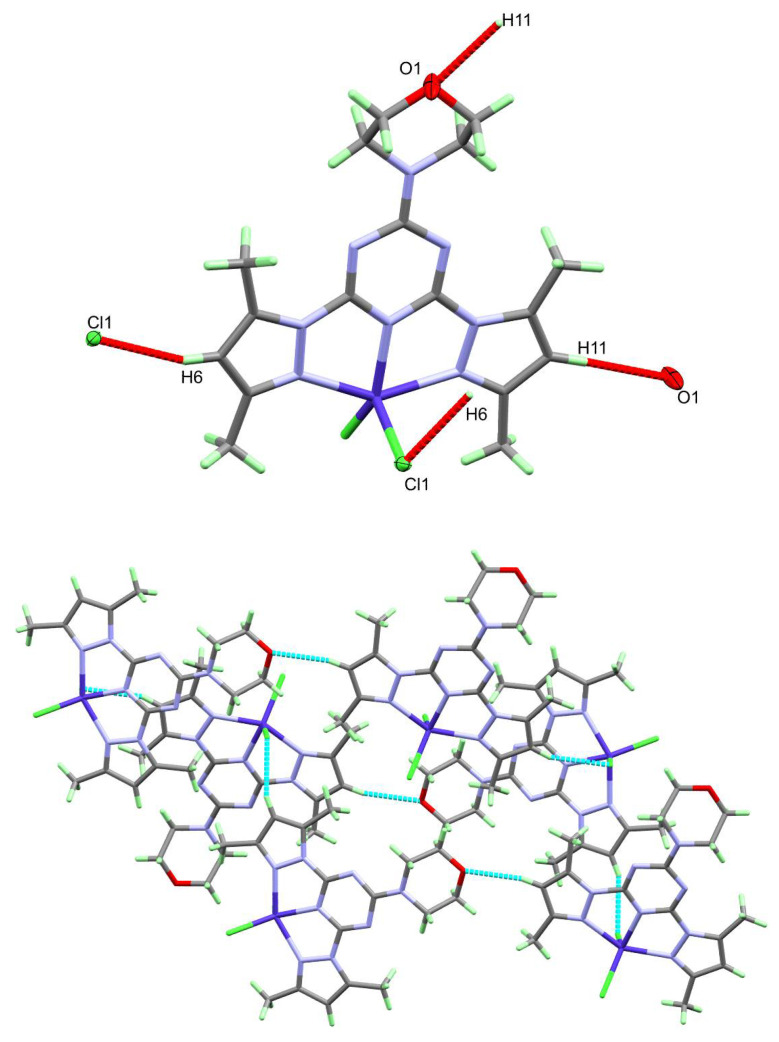
The hydrogen bond contacts (upper) and hydrogen bonding network (lower) in **1**.

The packing of complex molecules in **1** is controlled by O1…H11 and Cl1…H6 contacts shown in Figure 3 (upper part). The donor (D)-acceptor (A) distances are 3.503(3) and 3.550(2) Å for O1…H11 and Cl1…H6 hydrogen bond contacts, respectively (Table 3). Packing of complex units via C-H…Cl and C-H…O interactions is shown in Figure 3 (lower part).

#### 2.1.2. Structure Description of **[Co(^Pip^BPT)Cl_2_]** (2)

**Figure 4:**
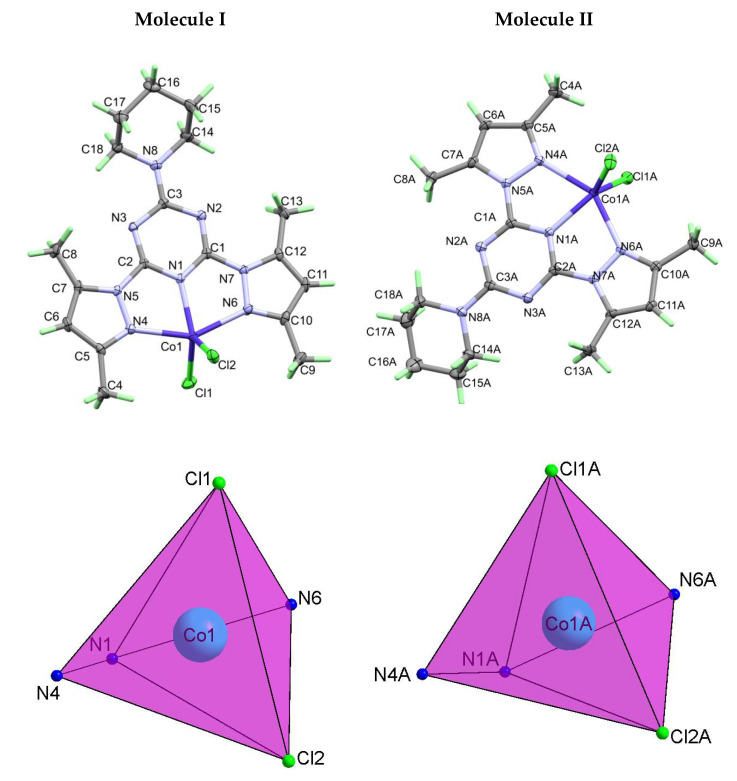
Structure with atom numbering (upper) and the distorted square pyramidal (lower) of **2**.

The **[Co(^Pip^BPT)Cl_2_]** complex (**2**) crystallized in the less symmetric triclinic crystal system and P-1 space group with Z = 4 and two molecular units as an asymmetric formula. In both units, the Co(II) is penta-coordinated with CoN_3_Cl_2_ coordination sphere comprising one **^Pip^BPT** ligand chelating the Co(II) ion in a pincer fashion augmented with two Co-Cl bonds at almost equal distances (Figure 4). Generally, the bond distances and angles of the two asymmetric formulas are very similar (Table 2). The τ values are 0.42 and 0.37 for the two molecular units **I** and **II** of **[Co(^Pip^BPT)Cl_2_]** complex indicating more distorted square pyramidal compared to complex **1**. It could be considered as an intermediate structure between square pyramidal and trigonal bipyramidal configurations. For molecule **I**, the angle between the *s*-triazine mean plane and each of the two pyrazolyl rings are 4.17 and 0.69° for the pyrazole moieties with lower and higher atom numbering, respectively. On the other hand, for molecule **II** the corresponding values are 4.71 and 7.76°, respectively.

**Figure 5:**
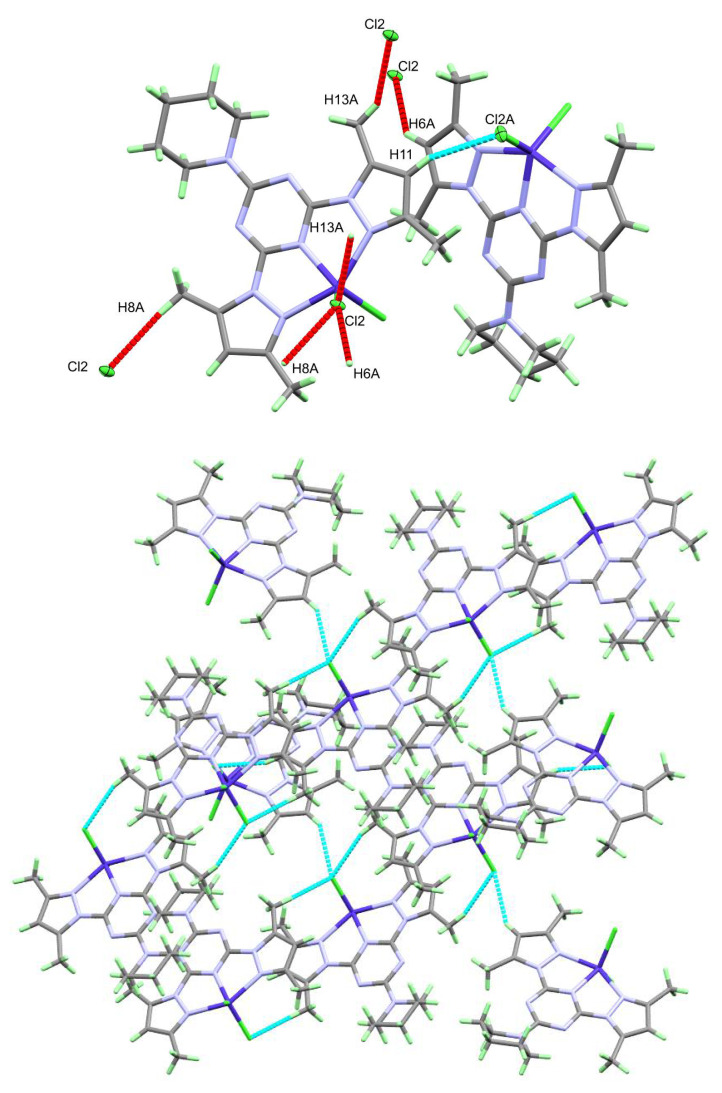
The hydrogen bond contacts (upper) and hydrogen bonding network (lower) in **2**.

The packing of complex molecules in **2** is controlled by Cl2…H6A, Cl2…H8A, and Cl2…H13A contacts shown in Figure 5 (upper part). The donor (D)-acceptor (A) distances are 3.524(2), 3.768(2) and 3.611(2) Å, respectively (Table 3). The hydrogen-bonding network in which the complex units are interconnected via C-H…Cl interactions as shown in Figure 5 (lower part).

### 2.2. Hirshfeld Topology Analyses

**Figure 6:**
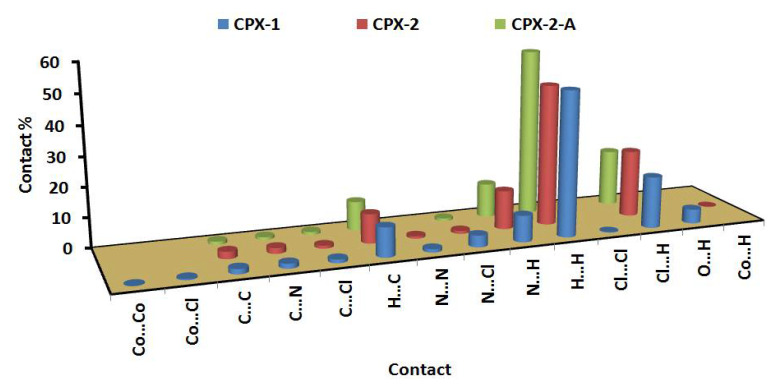
The important contacts and their percentages.

In order to further explore the different intermolecular contacts in the solid-state structure of the studied complexes, we employed Hirshfeld calculations (Figures S1–S3; Supplementary Data). Quantitative analysis results of all possible interactions are presented in Figure 6.

**Figure 7:**
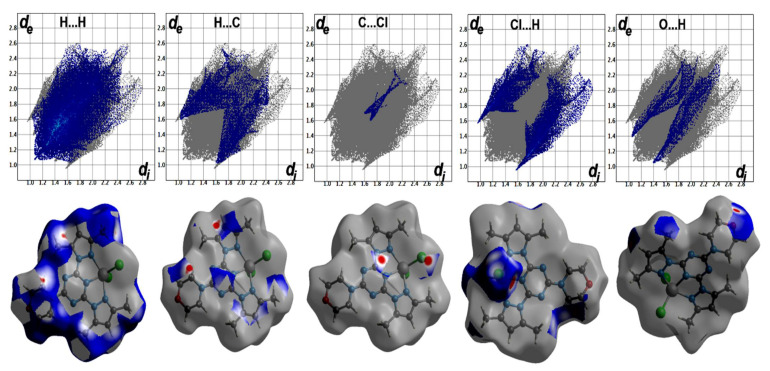
Hirshfeld surfaces of complex **1**.

**Figure 8:**
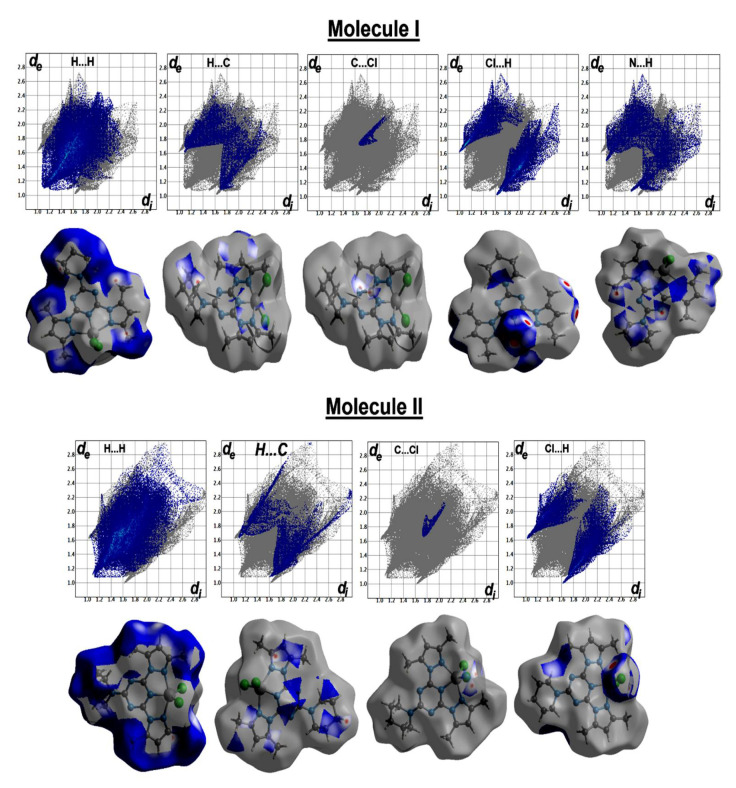
Hirshfeld surfaces of complex **2**.

The percentages of the H…H contacts are 48.8% and 47.7–56.1% from the whole contacts detected in **[Co(^Morph^BPT)Cl_2_]** and **[Co(^Pip^BPT)Cl_2_]**, respectively, while the Cl…H contact percentages are 17.4 and 18.9–22.5%, respectively. It is clear from the decomposed fingerprint and d_norm_ maps that these interactions have the characteristics of short contacts (Figure 7 and Figure 8). The shortest intermolecular contacts are listed in Table 4. In complex **1**, the packing is controlled by O…H and Cl…H hydrogen bonds as well as C-H…π and Cl…C (anion-π stacking) interactions. The latter belongs to the interaction between the coordinated chloride anion Cl2 and the C1 atom from the electron-deficient *s*-triazine moiety. In complex **2**, the packing is also dominated by short Cl…H, C-H…π and Cl…C (anion-π stacking) interactions. It is noted that the Cl2…C1 (3.297 Å) in complex **1** is significantly shorter than the Cl2A…C3 (3.407 Å) and Cl2A…C2 (3.412 Å) interactions in complex **2**. The H…C(π-system) are in the range of 2.642–2.751 and 2.722–2.727 Å in complexes **1** and **2**, respectively. Also, the C…C/C…N contacts having longer distances than the vdWs radii sum of the interacting elements indicated weak π-π interactions.

### 2.3. FTIR Spectra

**Figure 9:**
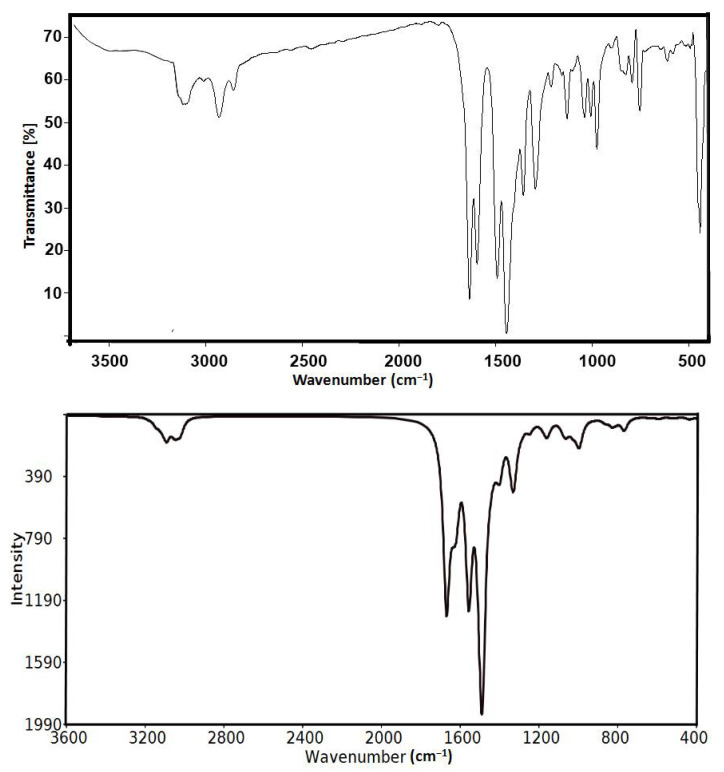
The experimental (upper) and calculated (lower) vibrational spectra of complex **2**.

The FTIR spectra of **[Co(^Morph^BPT)Cl_2_]** (**1**) and **[Co(^Pip^BPT)Cl_2_]** (**2**) showed some variations compared to the free ligands. The free **^Morph^BPT** and **^Pip^BPT** showed the C=N stretching modes at 1609 and 1603 cm^−1^, respectively. The corresponding values in complexes **1** and **2** showed significant shifts toward higher wavenumbers of 1633 and 1636 cm^−1^, respectively, due to the coordination of the Co(II) with the pincer ligand. Additionally, the ν_C = C_ modes in the free ligands were observed at 1529 and 1512 cm^−1^ for **^Morph^BPT** and **^Pip^BPT**, respectively. The ν_C = C_ modes are also significantly shifted to higher wave numbers of 1589 and 1597 cm^−1^ in **[Co(^Morph^BPT)Cl_2_]** (**1**) and **[Co(^Pip^BPT)Cl_2_]** (**2**), respectively. The presentation of the calculated vibrational spectra of complex **2** compared with the experimental FTIR spectra is shown in Figure 9. The results indicated two sharp bands at 1670.7 and 1558.1 cm^−1^ with relatively high intensity corresponding to the mixed C=N and C=C stretching vibrations. A comprehensive comparison of the experimental and calculated vibrational characteristics for complex **2** is provided in Table S1 (Supplementary Data). Generally, the calculated results are in fair agreement with the experimental results. For example, the calculated aromatic ν_C-H_ modes of the pyrazolyl moiety are calculated at 3272.4 cm^−1^ (exp. 3114.5 cm^−1^) while the asymmetric and symmetric aliphatic ν_C-H_ modes are calculated at 3146.7 cm^−1^ (exp. 2931.9 cm^−1^) and 3089.3–3021.0 cm^−1^ (exp. 2856.9 cm^−1^), respectively. The overestimations of the calculated vibrational frequencies compared to the experimental results are expected since the calculation was performed for a single molecule in the gas phase and hence neglects the anharmonicity present in the real system.

### 2.4. Electronic Spectra

**Figure 10:**
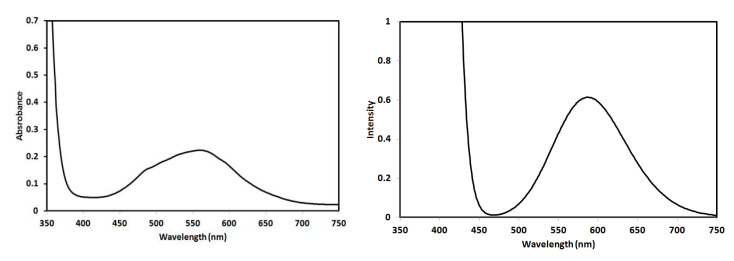
The experimental (left) and calculated (right) electronic spectra of complex **2**.

The electronic spectra of 4 × 10^−3^ M solution of complex **2** were recorded in ethanol as solvent. The experimentally observed UV-Vis spectra along with the simulated electronic spectra calculated using the TD-DFT method for complex **2** are shown in Figure 10. The recorded electronic spectra showed a broad spectral band at 564 nm, which was calculated at 589.9 nm.

**Figure 11:**
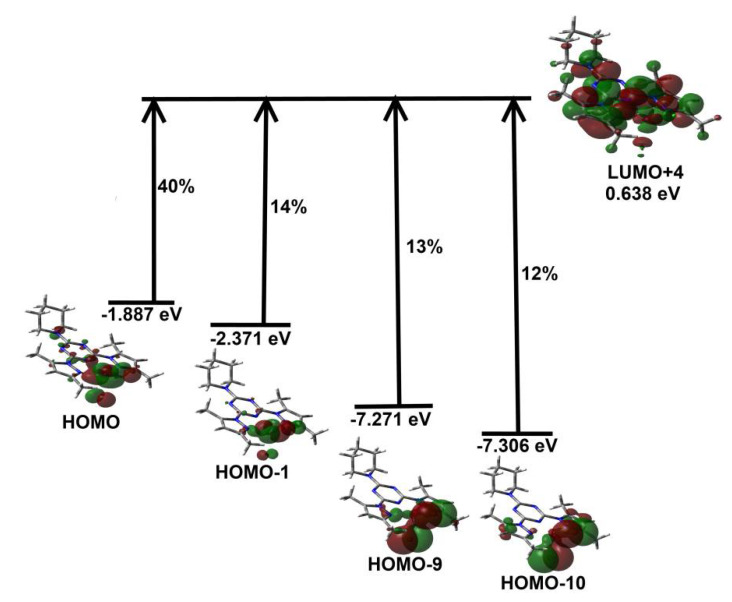
Origin of the electronic spectral band observed in ethanol for complex **2**.

In order to assign the origin of this electronic spectral band, the calculated excited and ground states included in this spectral band are shown in Figure 11. The band observed in the visible region could be assigned to electronic transitions from HOMO, HOMO-1, HOMO-9, and HOMO-10 as ground states to LUMO+4 as an excited state where all are β-type orbitals. This electronic transition could be described as mainly metal-ligand (**^Pip^BPT**) charge transfer-based transition.

### 2.5. Antimicrobial Activity

The biological activity of the free ligands (**^Morph^BPT** and **^Pip^BPT**), as well as the Co(II) complexes **[Co(^Morph^BPT)Cl_2_]** (**1**) and **[Co(^Pip^BPT)Cl_2_]** (**2**), were evaluated against *S. aureus* and *B. subtilis* as Gram-positive bacteria, *E. coli* and *P. vulgaris* as Gram-negative bacteria and two fungi (*A. fumigatus* and *C. albicans*). Minimum inhibition zone diameters were determined for the studied compounds (10 mg/mL) and the results are listed in Table 5.

The results shown in Table 5 indicated that the free ligands have no antimicrobial activity against all the studied microbes at the applied concentration (10 mg/mL) except **^Pip^BPT,** which is active only against the Gram-positive bacteria *B. subtilis* (13 mm). In contrast, the Co(II) complexes showed interesting antibacterial activities. Complex **1** is active against the two tested Gram-positive bacteria (*S. aureus* (20 mm) and *B. subtilis* (24 mm)) and one Gram-negative bacteria (*E. coli* (16 mm)). On the other hand, complex **2** showed significant antibacterial activities against all the studied bacteria strains with inhibition zone diameters ranging from 15 mm (*E. coli*) to 30 mm (*P. vulgaris*). An additional observation that could be concluded from these results; complex **2** has better antibacterial activity against *P. vulgaris* (30 mm) and very close antibacterial activities against *B. subtilis* (26 mm) compared to control (gentamycin: 27 mm). Both complexes showed no antifungal activity against the two tested fungi at the experimental conditions. The results indicated that the synthesized Co(II) complexes are promising antibacterial agents rather than antifungal agents.

Moreover, the minimum inhibitory concentrations (MIC) in μg/mL were determined and the results are depicted in Table 6. The results are in accord with our observations. The MIC values are the lowest for complex **2** against *B. subtilis*, *P. vulgaris*, and *S. aureus* indicated potent activities against these microbes. It is also more potent (complex **2**; 39 μg/mL) than **^Pip^BPT** against *B. subtilis* (87 μg/mL). Complex **1** has lower potency against the studied bacteria with higher MIC values ranging from 156–625 μg/mL.

### 2.6. Antioxidant Activity

**Figure 12:**
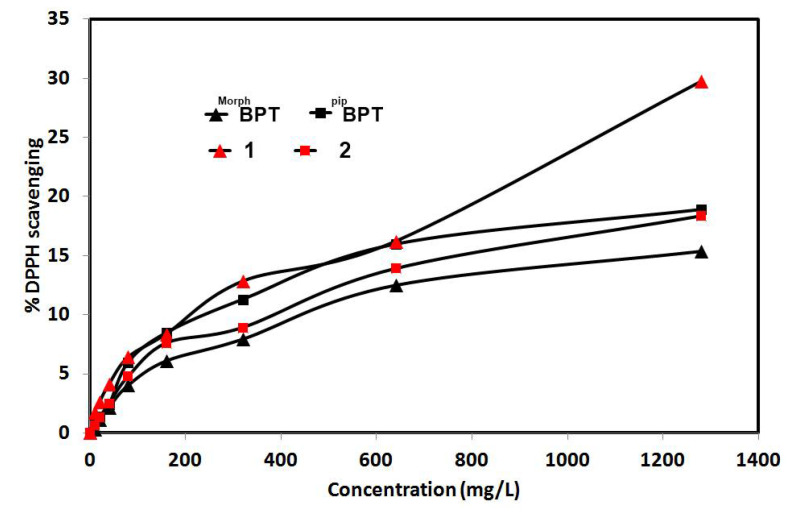
The antioxidant activity of the studied compounds.

The DPPH free radical scavenging assay enabled us to determine the antioxidant activity of the studied complexes compared to the free ligands. The detailed results are tabulated in Tables S2–S5 (Supplementary Data) and summarized graphically in Figure 12. Although the results showed that the studied systems have weak to moderate antioxidant activity, especially for the free ligands and complex **2,** but the most significant conclusion is that complex **1** has improved antioxidant activity compared to the free ligand **^Morph^BPT** while the antioxidant activity of **^Pip^BPT** and its **[Co(^Pip^BPT)Cl_2_]**; **2** are comparable indicating that varying the substituent at the *s*-triazine core of the functional ligand have a significant impact on the antioxidant activity of this class of Co(II) complexes.

## 3. Materials and Methods

Chemicals were purchased from Sigma-Aldrich Company (Chemie GmbH, 82024 Taufkirchen, Germany). The CHN analyses were determined using a Perkin-Elmer 2400 instrument (PerkinElmer, Inc., 940 Winter Street, Waltham, MA, USA). Cobalt content was determined using Shimadzu atomic absorption spectrophotometer (AA-7000 series, Shimadzu, Ltd., Kyoto, Japan). An Alpha Bruker spectrophotometer (Billerica, MA, USA) was used to measure the FTIR spectra in KBr pellets (Figures S4 and S5, Supplementary Data). The FTIR spectra were recorded in the range of 4000–400 cm^−1^ at a spectral resolution of 2 cm^−1^ and with 40 scans. The UV-Vis electronic spectra were recorded in ethanol using Pg instruments T80+ spectrophotometer (Alma Park, Wibtoft, UK). The melting points were ascertained in open capillary tubes using a Gallenkamp melting point apparatus (Sigma-Aldrich Chemie GmbH, Taufkirchen, Germany) and were uncorrected.

### 3.1. Syntheses of **[Co(^Morph^BPT)(Cl)_2_]**; (1) and **[Co(^Pip^BPT)(Cl)_2_]**; (2)

The ligands were prepared following the same method reported by us [53]. Details regarding the ligand preparations were given in (Supplementary Material Method S1, Figures S6 and S7).

A 10 mL methanolic solution of 0.5 mmol of the functional *s*-triazine chelate was added to 10 mL aqueous solution of the CoCl_2_ (64.9 mg, 0.5 mmol). Purple color crystals of the titled complexes were obtained after five days.

Yield; C_17_H_22_Cl_2_CoN_8_O (**1**) 86%; mp > 360 °C (dec). Anal. Calc. C, 42.16; H, 4.58; N, 23.14; Co, 12.17%. Found: C, 41.93; H, 4.49; N, 23.01; Co, 12.05%. IR (KBr, cm^−1^): 3090, 2983, 2924, 2859, 1633, 1589, 1499, 1448, 1067, 1020.

Yield; C_18_H_24_Cl_2_CoN_8_ (**2**) 81%; mp > 360 °C (dec). Anal. Calc. C, 44.83; H, 5.02; N, 23.23; Co, 12.22%. Found: C, 44.98; H, 4.94; N, 23.09; Co, 12.10%. IR (KBr, cm^−1^): 3115, 2932, 2857, 1636, 1597, 1493, 1444, 1041, 1008.

### 3.2. Crystal Structure Determination

A Bruker D8 Quest diffractometer was used to determine the crystal structures of complexes **1**–**2** with the aid of SHELXTL and SADABS programs [54,55,56]. Refinement and crystal details were given in Table 1. Hirshfeld calculations were performed using the Crystal Explorer 17.5 program [57,58,59,60,61,62].

### 3.3. Antimicrobial Studies

The antimicrobial activity of the free **^Morph^BPT** and **^Pip^BPT** ligands, as well as the corresponding Co(II) complexes, against two Gram-positive bacteria (*S. aureus* (ATCC 25923) and *B. subtilis* (RCMB015(1)NRR LB-543)), two Gram-negative bacteria (*E. coli* (ATCC 25922) and *P. vulgaris* (RCMB 004(1)ATCC 13315)), and two fungi (*A. fumigatus* (RCMB 002008) and *C. albicans* (RCMB 005003(1) ATCC 10231)). Minimum inhibition zone diameters at 10 mg/mL of the studied compounds, as well as the minimum inhibitory concentrations (MIC), were determined against these microbes [53]. Gentamycin and ketoconazole were used as standard antibacterial and antifungal agents, respectively. More details are found in (Method S2 Supplementary Data).

### 3.4. Antioxidant Activity

The antioxidant activity of complexes **1** and **2** was determined at the Regional Center for Mycology and Biotechnology (RCMB) at the Al-Azhar University by the DPPH free radical scavenging assay in triplicate and average values were considered [63,64]. More details regarding the bio-experiments are found in (Method S3 Supplementary Data).

### 3.5. DFT Calculations

The structure of complex **2** was optimized in the gas phase using the B3LYP method employing 6–31G(d,p) for nonmetal atoms and LANL2DZ for Co [65] with the aid of Gaussian 09 software [66]. All frequency results are positive and no imaginary frequency indicating real minima. The gas-phase optimized structure was used as the input for simulating the structure in ethanol as solvent followed by TD-DFT calculations in the same solvent in order to simulate and assign the experimentally observed UV-Vis spectra [67,68].

## 4. Conclusions

Two penta-coordinated Co(II) complexes with *bis*-pyrazolo-*s*-triazine pincer ligands bearing morpholino (**^Morph^BPT**) and piperidino (**^Pip^BPT**) substituents were synthesized and their structure aspects were analyzed using single-crystal X-ray diffraction and Hirshfeld analysis. The mononuclear **[Co(^Morph^BPT)Cl_2_]**; **1** and **[Co(^Pip^BPT)Cl_2_]**; **2** pincer complexes have a similar coordination environment comprising a tridentate functional ligand and two coordinated chloride ions. Complex **2** has higher potency against all the studied bacteria (except *E. coli*) than complex **1**. In addition, the antioxidant activity of complex **1** is higher than **^Morph^BPT,** while both **2** and **^Pip^BPT** have comparable results. These outcomes shed light on the importance of the nature of the substituent on the *s*-triazine ring of the coordinated functional ligand on the antioxidant activity. The design of *s*-triazine ligands carrying different substituents could improve the antioxidant activity, which is one of our future perspectives.

## Figures and Tables

**Table 1:** Crystal data and structure refinement for the studied complexes.

Compound	[Co(^Morph^BPT)Cl_2_] (1)	[Co(^pip^BPT)Cl_2_] (2)
Empirical formula	C_17_ H_22_ Cl_2_ Co N_8_ O	C_18_ H_24_ Cl_2_ Co N_8_
Formula weight	484.25 g/mol	482.28 g/mol
Temperature	121(2) K	121(2) K
Wavelength	0.71073 Å	0.71073 Å
Crystal system	Monoclinic	Triclinic
Space group	C2/c	P-1
Unit cell dimensions	a = 17.241(5) Å	a = 11.094(3) Å
	b = 12.832(3) Å	b = 12.785(3) Å
	c = 20.165(7) Å	c = 16.527(4) Å
	α = 90°	α = 97.125(6)°
	β = 109.687(9)°	β = 103.496(6)°
	γ = 90°	γ = 102.933(6)°
Volume	4200.(2) Å^3^	2182.8(10) Å^3^
Z	8	4
Density (calculated)	1.531 g/cm^3^	1.468 g/cm^3^
Absorption coefficient	1.098 mm^−1^	1.053 mm^−1^
F(000)	1992	996
Crystal size	0.14 × 0.21 × 0.25 mm^3^	0.20 × 0.27 × 0.32 mm^3^
Theta range for data collection	2.51 to 25.34°	2.21 to 25.32°
Index ranges	−20 ≤ h ≤ 20	−13 ≤ h ≤ 13
	−15 ≤ k ≤ 13	−15 ≤ k ≤ 15
	−24 ≤ l ≤ 24	−19 ≤ l ≤ 19
Reflections collected	14,323	35,520
Independent reflections	3834 [R(int) = 0.0293]	7949 [R(int) = 0.0373]
Completeness to theta	99.60%	99.70%
Refinement method	Full-matrix least-squares on F^2^	
Data/restraints/parameters	3834/0/267	7949/0/532
Goodness-of-fit on F2	1.033	1.041
Final R indices (I > 2sigma(I))	R1 = 0.0250, wR2 = 0.0581	R1 = 0.0246, wR2 = 0.0548
R indices (all data)	R1 = 0.0310, wR2 = 0.0616	R1 = 0.0324, wR2 = 0.0584
Extinction coefficient	0.00125(12)	0.0036(3)
Largest difference peak and hole	0.342 and −0.353	0.315 and −0.272
CCDC	20805833	20805834

**Table 2:** Bond lengths (Å) and angles (°) for complexes **1** and **2**.

[Co(^Morph^BPT)Cl_2_] (1)			[Co(^Pip^BPT)Cl_2_] (2)		
Co1-N1	2.0387(15)	Co1-N1	2.0291(15)	Co1A-N1A	2.0325(15)
Co1-N4	2.2008(15)	Co1-N4	2.1829(15)	Co1A-N4A	2.2023(15)
Co1-N6	2.2304(16)	Co1-N6	2.1935(15)	Co1A-N6A	2.2184(15)
Co1-Cl2	2.2718(7)	Co1-Cl2	2.2766(7)	Co1A-Cl2A	2.2667(6)
Co1-Cl1	2.2968(7)	Cl1-Co1	2.2822(7)	Co1A-Cl1A	2.2686(8)
N1-Co1-N4	74.01(6)	N1-Co1-N4	73.73(6)	N1A-Co1A-N4A	73.83(6)
N1-Co1-N6	73.07(5)	N1-Co1-N6	74.23(6)	N1A-Co1A-N6A	73.72(6)
N4-Co1-N6	146.87(5)	N4-Co1-N6	147.94(5)	N4A-Co1A-N6A	147.43(5)
N1-Co1-Cl2	138.89(4)	N1-Co1-Cl2	119.81(4)	N1A-Co1A-Cl2A	115.61(5)
N4-Co1-Cl2	102.34(4)	N4-Co1-Cl2	97.95(4)	N4A-Co1A-Cl2A	100.41(4)
N6-Co1-Cl2	99.89(4)	N6-Co1-Cl2	98.88(4)	N6A-Co1A-Cl2A	95.97(4)
N1-Co1-Cl1	109.63(5)	N1-Co1-Cl1	122.66(4)	N1A-Co1A-Cl1A	124.96(4)
N4-Co1-Cl1	97.96(4)	N4-Co1-Cl1	100.29(4)	N4A-Co1A-Cl1A	95.76(4)
N6-Co1-Cl1	96.42(4)	N6-Co1-Cl1	95.81(4)	N6A-Co1A-Cl1A	100.40(4)
Cl2-Co1-Cl1	111.40(3)	Cl2-Co1-Cl1	117.51(2)	Cl2A-Co1A-Cl1A	119.43(2)

**Table 3:** Geometric parameters of the hydrogen bonds [Å and °] in complexes **1** and **2**.

**1**				
C6-H6…Cl1 ^i^	0.95	2.65	3.550(2)	158
C11-H11…O1 ^ii^	0.95	2.58	3.503(3)	165
^i^ 1/2-x,-1/2+y,3/2-z; ^ii^ 1/2+x,3/2-y,1/2+z				
**2**				
C6A-H6A…Cl2 ^i^	0.95	2.74	3.524(2)	140
C8-H8A…Cl2 ^ii^	0.98	2.82	3.768(2)	162
C13-H13A…Cl2 ^iii^	0.98	2.76	3.611(2)	146

^i^ 1+x,y,z; ^ii^ -x,-y,1-z; ^iii^ 1-x,1-y,1-z.

**Table 4:** The most important contacts in complexes **1** and **2**.

1		2	
Contact	Distance	Contact	Distance
C6…H14B	2.745	C5A…H16D	2.727
C7…H14B	2.642	C10A…H17A	2.722
C8…H14B	2.718	Cl2A…C3	3.407
C3…H4A	2.751	Cl2A…C2	3.412
Cl2…C1	3.297	Cl2…H6A	2.642
Cl1…H6	2.527	Cl1A…H4C	2.780
O1…H11	2.447	Cl2A…H4A	2.797
H4C…H15A	2.223	Cl2…H13A	2.670
C6…C2	3.495	Cl2…H8A	2.724
C6…N7	3.390	Cl1...H18B	2.816
		H9A…H13B	2.294
		C11…N4A	3.347
		C11…N5A	3.341
		N2…H8B	2.578

**Table 5:** Inhibition zone diameters of the studied compounds and control against different microbes ^a^.

Micorbe	^Morph^BPT	1	^Pip^BPT	2	Control
*A. fumigatus*	-	-	-	-	17 ^a^
*C. albicans*	-	-	-	-	20 ^a^
*S. aureus*	-	20	-	18	25 ^b^
*B. subtilis*	-	24	13	26	27 ^b^
*E. coli*	-	16	-	15	30 ^b^
*P. vulgaris*	-	-	-	30	27 ^b^

^a^ Ketoconazole and ^b^ Gentamycin.

**Table 6:** MIC values (μg/mL) for the studied compounds.

Micorbe	^Morph^BPT	1	^Pip^BPT	2
*A. fumigatus*	-	-	-	-
*C. albicans*	-	-	-	-
*S. aureus*	-	156	-	20
*B. subtilis*	-	312	78	39
*E. coli*	-	625	-	1250
*P. vulgaris*	-	-	-	20

## Data Availability

The data presented in this study are available on request from the corresponding author.

## Supplementary Materials

The following are available online at; Method S1: General method for the synthesis of bis-pyrazolo-*s*-triazine derivatives. Method S2: Antimicrobial studies. Method S3: DPPH radical scavenging activity. Figure S1 Hirshfeld surfaces of complex 1. Figure S2 Hirshfeld surfaces of complex 2 (molecule I). Figure S3 Hirshfeld surfaces of complex 2 (molecule II). Figure S4 FTIR spectra of the free ligand **^Morph^BPT** (upper) and its Co(II) complex **[Co(^Morph^BPT)Cl_2_]**; 1 (lower). Figure S5 FTIR spectra of the free ligand **^Pip^BPT** (upper) and its Co(II) complex **[Co(^Pip^BPT)Cl_2_]**; 2 (lower). Figure S6: ^1^H NMR and ^13^C NMR of compound **^Morph^****BPT**. Figure S7: ^1^H NMR and ^13^C NMR of compound **^Pip^****BPT**. Table S1 Calculated and experimental vibrational characteristics for complex 2. Table S2 Evaluation of antioxidant activity using DPPH scavenging assay for **^Morph^BPT**. Table S3 Evaluation of antioxidant activity using DPPH scavenging assay for **[Co(^Morph^BPT)Cl_2_]**; 1. Table S4 Evaluation of antioxidant activity using DPPH scavenging assay for **^Pip^BPT**. Table S5 Evaluation of antioxidant activity using DPPH scavenging assay for **[Co(^Pip^BPT)Cl_2_]**; 2.

## Sample Availability

Samples of the compounds are not available from the authors.
