# Supplementary material for: Syntheses and Structural Investigations of Penta-Coordinated Co(II) Complexes with Bis-Pyrazolo-S-Triazine Pincer Ligands, and Evaluation of Their Antimicrobial and Antioxidant Activities
Source: Molecules. 2021 Jun 14;26(12):3633. doi: 10.3390/molecules26123633 (PMC8232275; doi:10.3390/molecules26123633)
Supplement: Supplementary file 1 [file molecules-26-03633-s001.zip › molecules-1221486-supplementary.pdf]

Syntheses and Structural Investigations of Penta-Coordinated Co(II) Complexes with *Bis*-Pyrazolo-*S*-Triazine Pincer Ligands, and Evaluation of Their Antimicrobial and Antioxidant Activities

Saied M. Soliman [1,*], Raghdaa A. Massoud [1], Hessa H. Al-Rasheed [2] and Ayman El-Faham [1,2,*]

[1] Department of Chemistry, Faculty of Science, Alexandria University, P.O. Box 426, Ibrahimia,
Alexandria 21321, Egypt; raghdaamassoud@yahoo.com

[2] Department of Chemistry, College of Science, King Saud University, P.O. Box 2455, Riyadh 11451, Saudi Arabia; halbahli@ksu.edu.sa

* Correspondence: saied1soliman@yahoo.com (S.M.S.), Tel.: +20-111-1361-059; aelfaham@ksu.edu.sa (A.E.-F.), Tel.: +966-114-673-195

Method S1: General method for the synthesis of bispyrazolo-s-triazine derivatives

1,3,5-Triazine dihydrazino derivative [45] (10 mmol) was dissolved in 20 mL DMF and then acetylacetone (25 mmol) was added, followed by addition of triethylamine (15 mmol) in 10 mL DMF with stirring at room temperature. The reaction mixture was refluxed for 6–8 h at 60 ºC. The progress of the reaction was monitored by TLC using ethylacetate-hexane (4:6). The solution was allowed to cool to room temperature, and then ice cold water was added with continuous stirring. The reaction mixture was kept in an ice bath for 2–3 h, and the product was collected by filtration, washed with cold water (3×20 mL), and then dried under vacuum. The crude product was recrystallized from ethanol to afford the products in good yields.

4-(4,6-bis(3,5-dimethyl-1H-pyrazol-1-yl)-1,3,5-triazin-2-yl)morpholine; ^{Morp}**BPT**

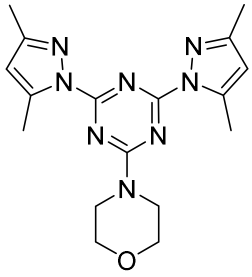

White crystals, mp = 187–188°C, in yield 72%; ^{1}H NMR ($CDCl_3$): δ 2.27 (s, 6H, $2CH_3$), 2.632 (s, 6H, $2CH_3$), 3.56 (t, 4H, J = 2.4 Hz, $2CH_2$), 3.86 (t, 4H, J = 4.4 Hz, $2CH_2$), 5.97 (s, 2H, 2CH) ppm; ^{13}C NMR ($CDCl_3$): δ 10.7, 12.9, 41.2, 63.4, 108.1, 140.5, 149.0, 160.5, 162.4 ppm.

2,4-bis(3,5-dimethyl-1H-pyrazol-1-yl)-6-(piperidin-1-yl)-1,3,5-triazine, ^{Pip}**BPT**

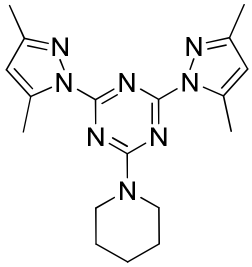

White crystals, mp = 141–142°C, in yield 73%; ^1H NMR ($CDCl_3$): δ 1.60 (m, 6H, $3CH_2$), 2.27 (s, 6H, $2CH_3$),2.62(s, 6H, $2CH_3$), 3.78 (t, 4H, , *J* = 4.4 Hz, $2CH_2$), 5.97 (s,2H, 2CH) ppm; ^{13}C NMR ($CDCl_3$): δ 13.5, 15.8, 24.3, 25.5, 45.1, 110.8, 143.3, 151.7, 163.5, 164.7 ppm.

Method S2: Antimicrobial studies

a) Tested pathogenic microbes

The antibacterial activity of the studied ligands and their Co(II) complexes were evaluated against two Gram positive bacteria ((*S. aureus* (ATCC 25923) and *B. subtilis* (RCMB015(1)NRR LB-543)), two Gram negatvie bacteria ((*E. coli* (ATCC 25922) and *P. vulgaris* (RCMB 004(1)ATCC 13315)) and two fungi ((*A. fumigatus* (RCMB 002008) and *C. albicans* (RCMB 005003(1) ATCC 10231)). Gentamycin was used as standard antibacterial agent. The samples maintained in Brain heart infusion (BHI) at 20°C; 300 mL of each stock–culture was added to 3 mL of BHI broth. Overnight cultures were kept for 24 h at 37 °C ± 1°C and the purity of cultures was checked after 24 h of incubation. After 24 h of incubation, bacterial suspension was diluted with sterile physiological solution, for the diffusion and indirect bioautographic tests, to 108 CFU/mL (turbidity = McFarland barium sulfate standard 0.5). In case of fungi *A. fumigatus* (RCMB 002008) and *C. albicans* (RCMB 005003(1) ATCC 10231), the used medium in antagonistic activity against tested fungi is Potato Dextrose Agar, where Fluconazole was used standard antifungal agent.

b) Agar well diffusion method

Synthetic compounds were prepared at concentration 10 mg/mL dissolved in DMSO as stock solutions. Preparation of sterilized Mueller Hinton agar plates seeded with tested pathogenic bacteria occurred. The wells are done by sterilized cork borer in size 6 mm and hence 200 μg of the synthetic compound was poured in each well comparably with DMSO as control. The plates were incubated at 37°C for 24 h. after incubation period; antimicrobial activity was determined by inhibition zones.

c) Minimum Inhibitory Concentration (MIC)

Different dilutions of the compounds are inoculated with tested pathogenic microbes. After incubation period of 96 well microplate, the results are measured using microplate reader. To determine at what level the MIC endpoint is established; subculture of test samples at different concentrations occurred in nutrient agar plates.

Method S3: DPPH Radical Scavenging Activity:

Freshly prepared (0.004%*w*/*v*) methanol solution of 2,2-diphenyl-1-picrylhydrazyl (DPPH) radical was prepared and stored at 10 ºC in the dark. A methanol solution of the test compound was prepared. A 40 uL aliquot of the methanol solution was added to 3 ml of DPPH solution. Absorbance measurements were recorded immediately with a UV-visible spectrophotometer [S1]. The decrease in absorbance at 515 nm was determined continuously, with data being recorded at 1 min intervals until the absorbance stabilized (16 min). The absorbance of the DPPH radical without antioxidant (control) and the reference compound ascorbic acid were also measured. All the determinations were performed in three replicates and averaged. The percentage inhibition (PI) of the DPPH radical was calculated according to the formula:

$$PI = [\{(AC- AT)/ AC\} \times 100] \quad (1)$$

Where *A*C = Absorbance of the control at t = 0 min and *A*T = absorbance of the sample+DPPH at t = 16 min [S2]

The 50% inhibitory concentration (IC_{50}), the concentration required to inhibit DPPH radical by 50%, was estimated from graphic plots of the dose response curve.

Reference :

[S1] Yen, G.C. and Duh, P.D. Scavenging effect of methanolic extracts of peanut hulls on free radical and active oxygen species, *J. Agric. Food. Chem.*, 1994, *42*, 629-632.

[S2] Al Zahrani, N.A.; El-Shishtawy, R.M.; Elaasser, M.M.; Asiri, A.M. Synthesis of novel chalcone-based phenothiazine derivatives as antioxidant and anticancer agents. *Molecules* 2020, *25*, 4566.

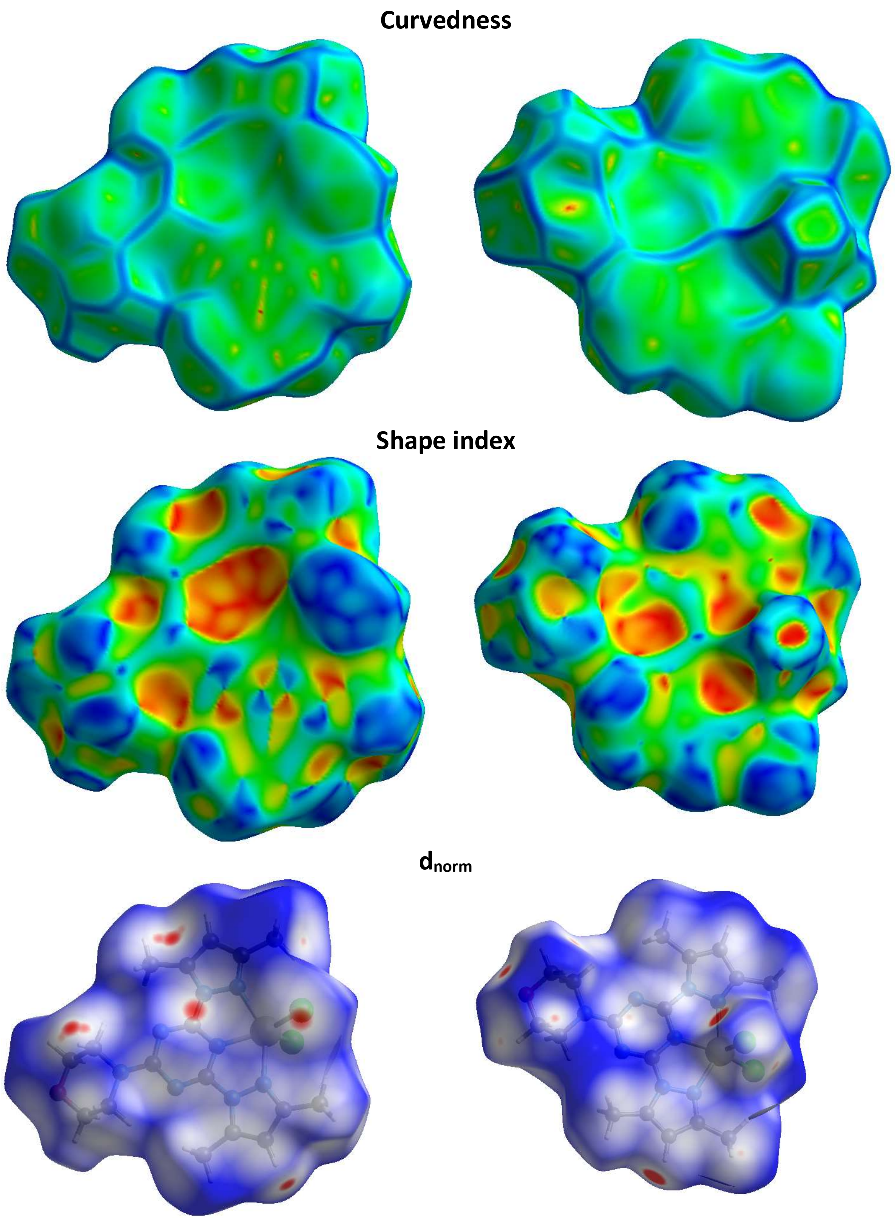

Figure S1 Hirshfeld surfaces of complex **1**.

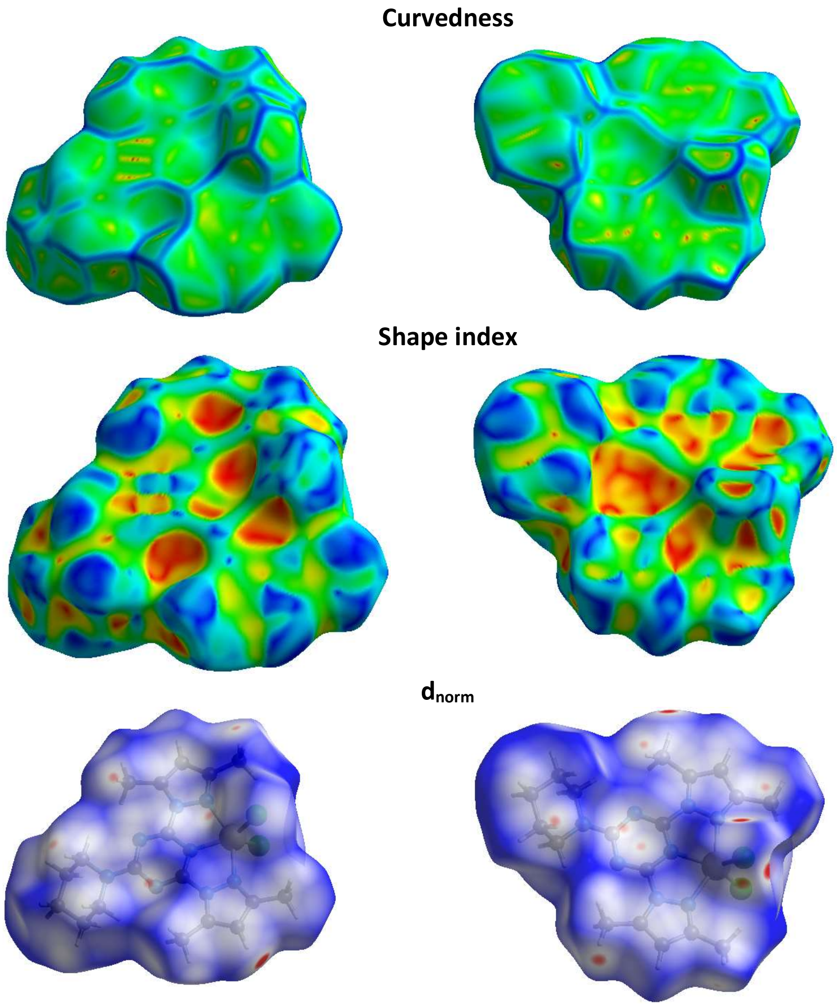

Figure S2 Hirshfeld surfaces of complex **2 (molecule I)**.

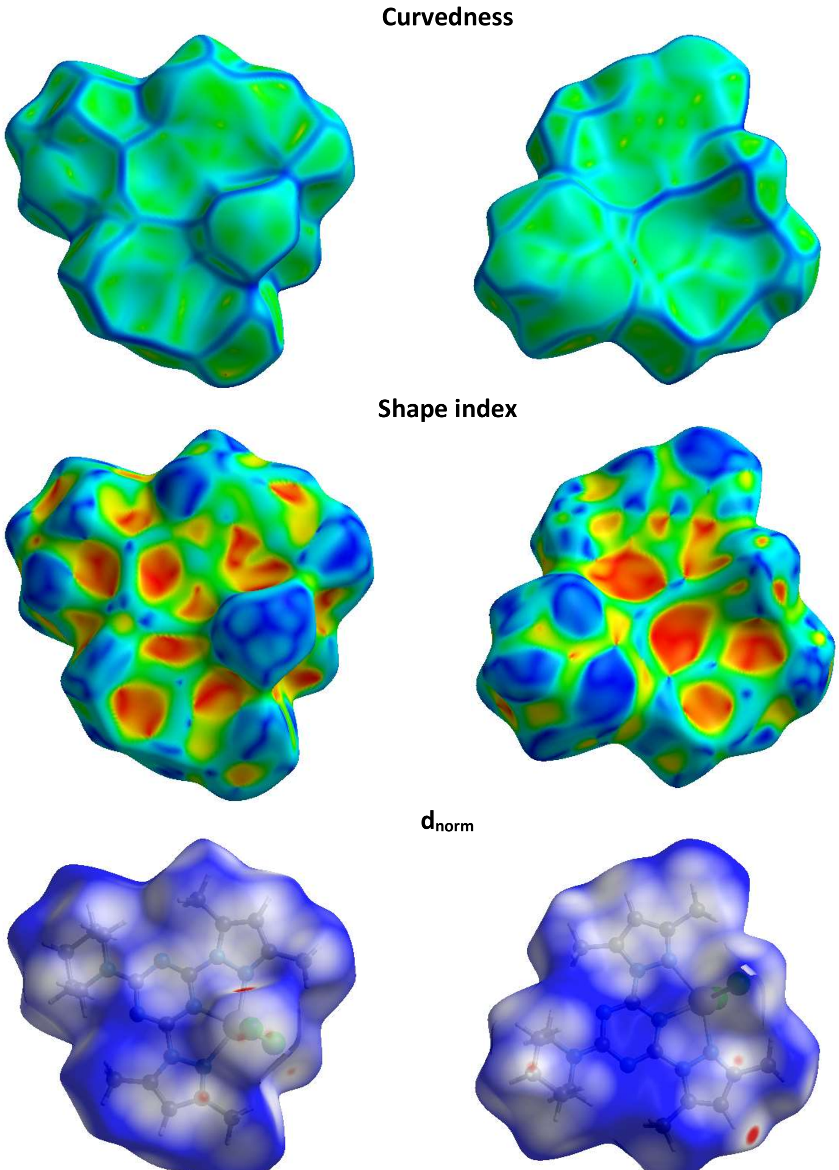

Figure S3 Hirshfeld surfaces of complex **2 (molecule II).**

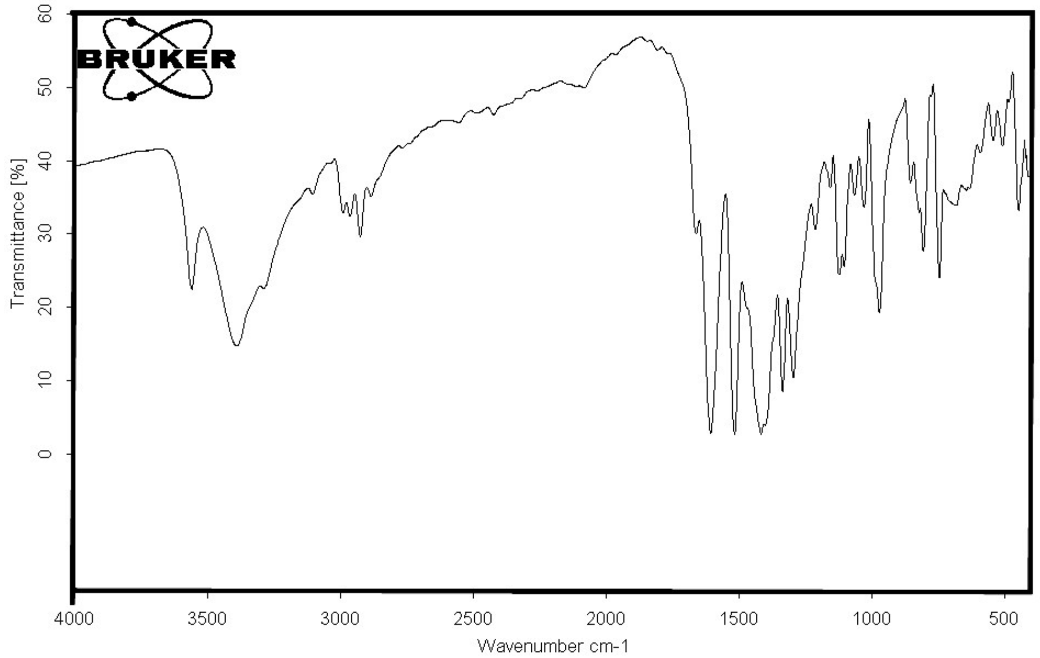

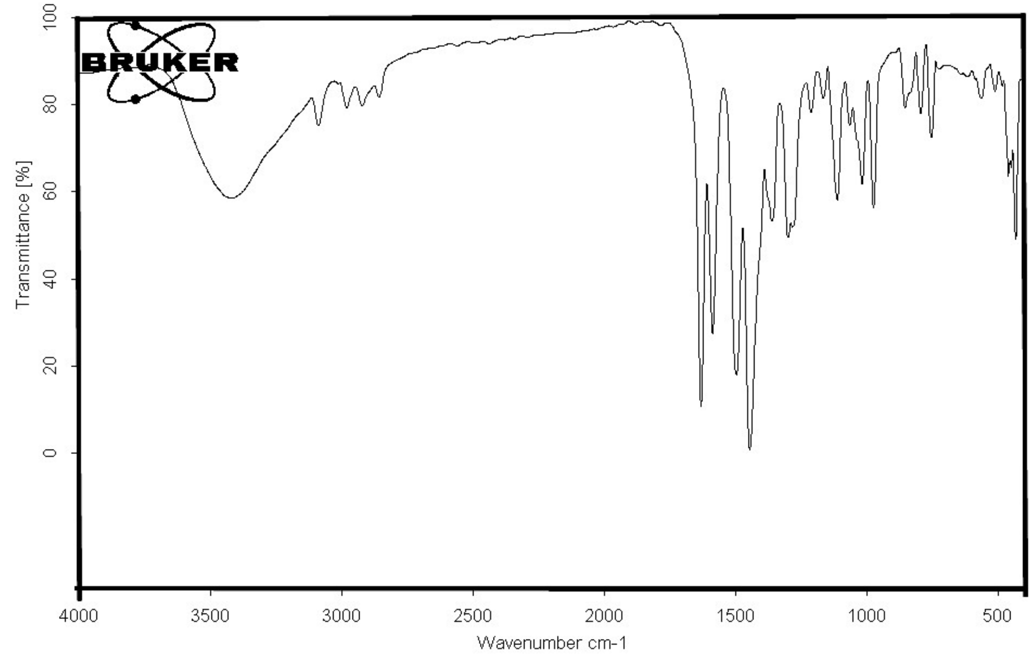

Figure S4 FTIR spectra of the free ligand [Morph]**BPT** (upper) and its Co(II) complex [Co([Morph]BPT)Cl_2]; **1** (lower).

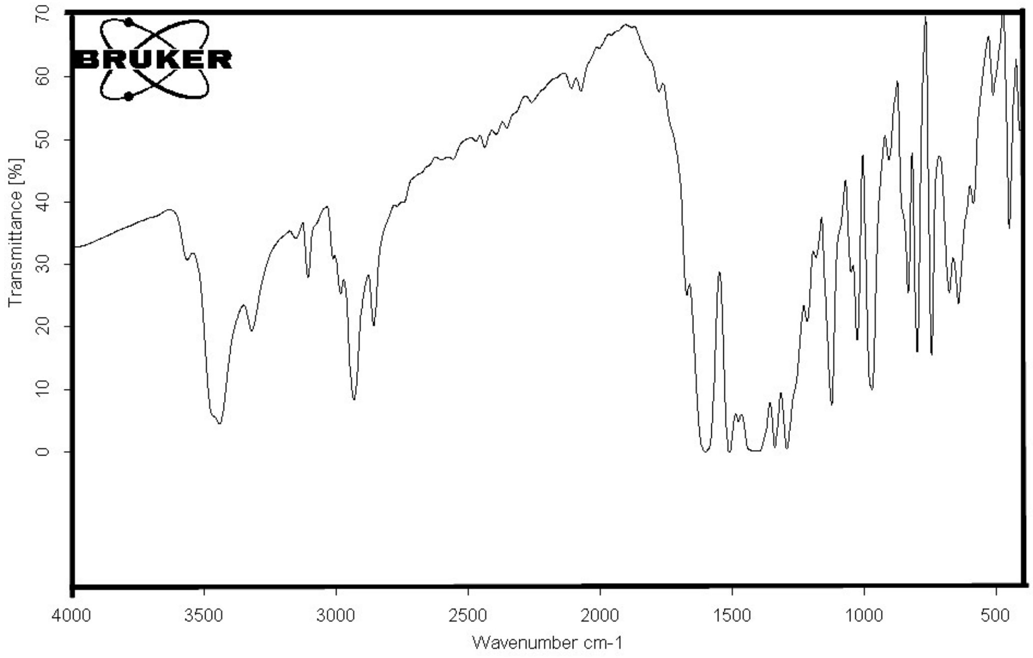

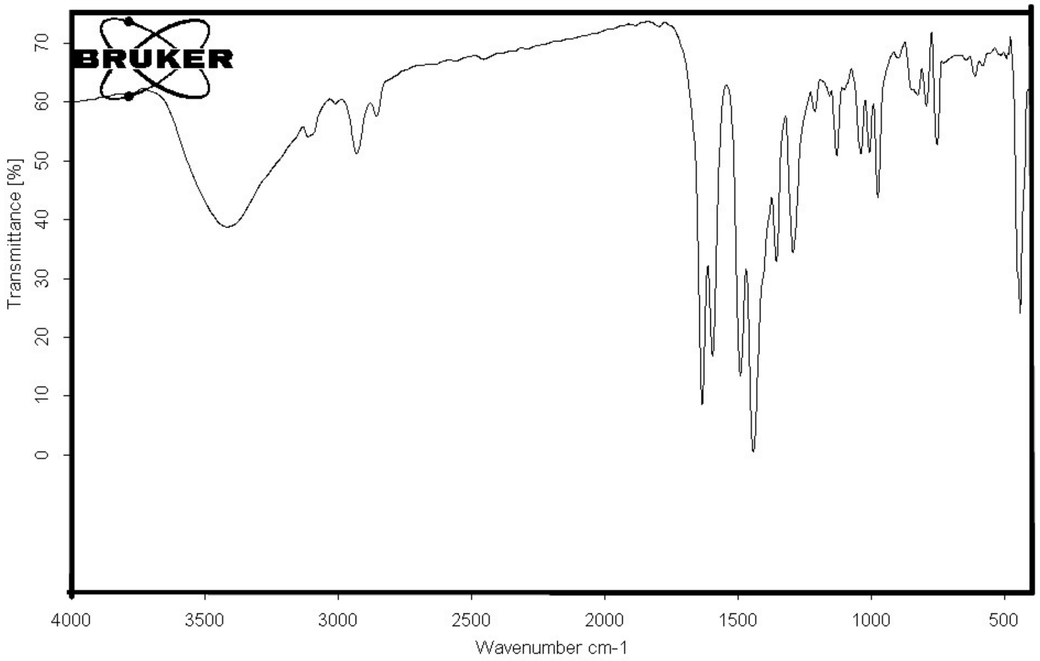

Figure S5 FTIR spectra of the free ligand **^{Pip}BPT** (upper) and its Co(II) complex **[Co(^{Pip}BPT)Cl_2]; 2** (lower).

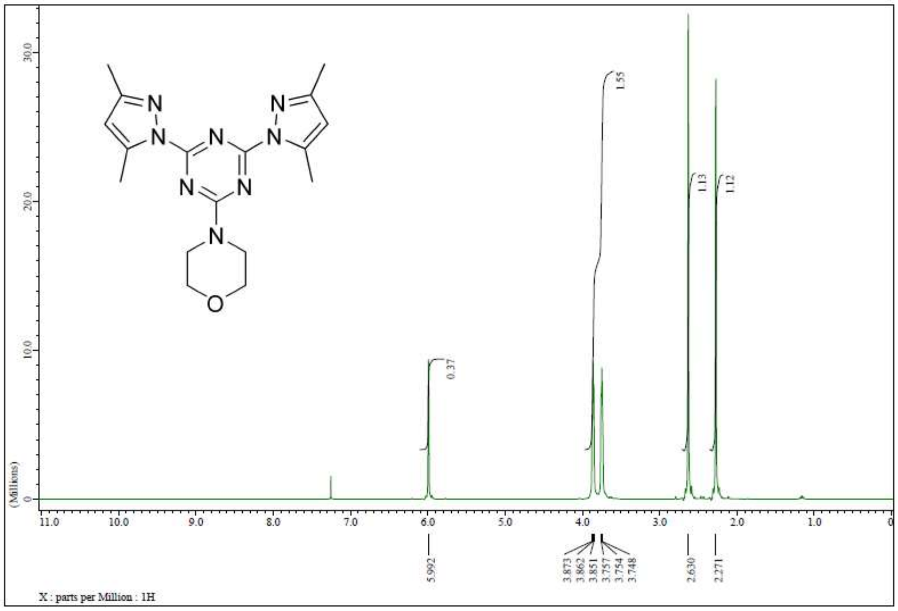

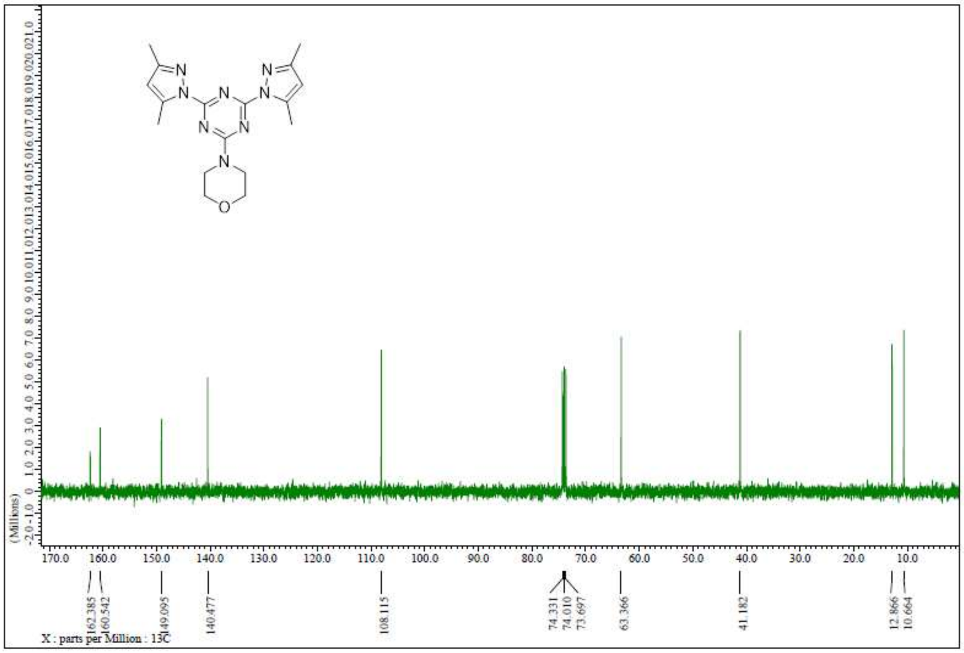

Figure S6: ^{1}H NMR and ^{13}C NMR of compound ^{Morph}**BPT**.

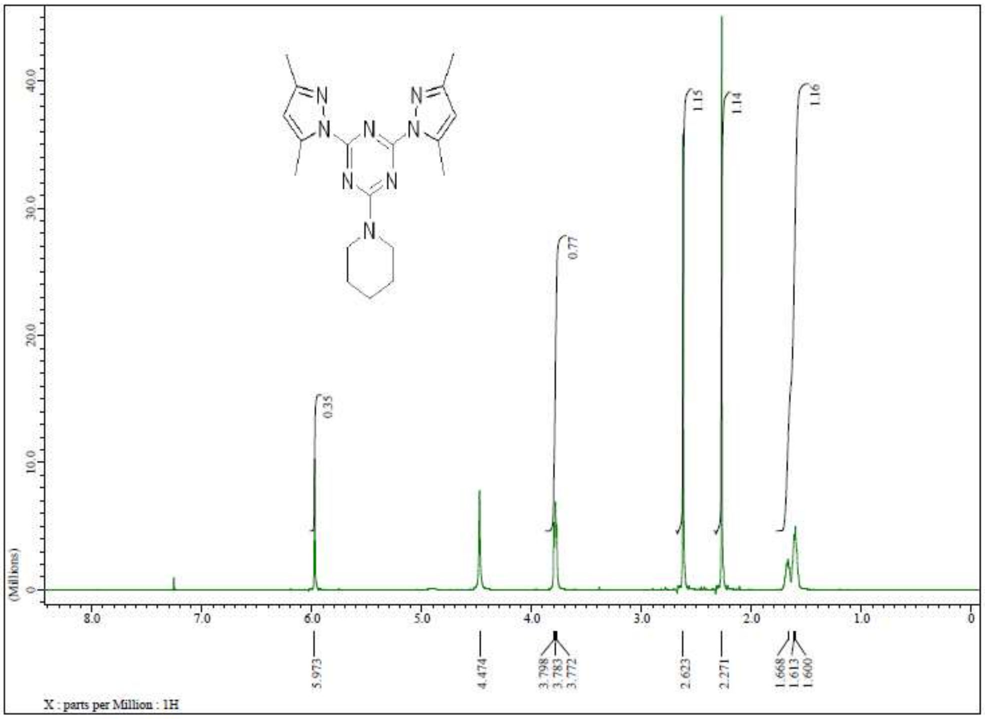

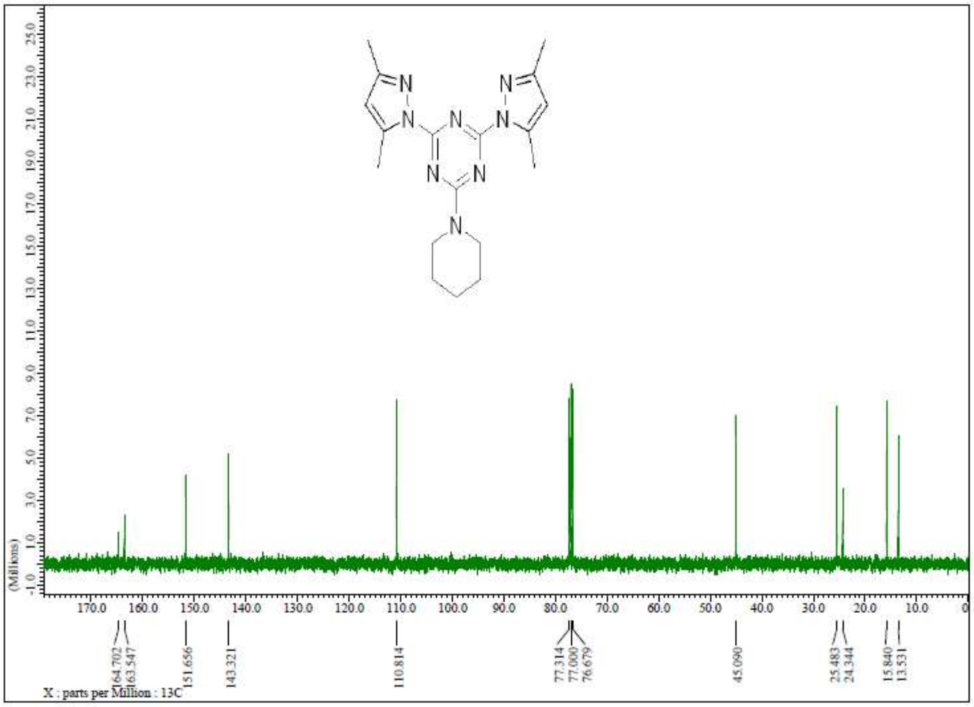

Figure S7: ^{1}H NMR and ^{13}C NMR of compound ^{Pip}**BPT**

Table S1 Calculated and experimental vibrational characteristics and their assignments for complex **2.**

ν_{calc} (cm^{-1})	ν_{exp} (cm^{-1})	Assignment[a]
3272.4	3114.5	$\nu_{C\text{-}H(aromatic)}$
3146.7	2931.9	Asym $\nu_{C\text{-}H(Aliphatic)}$
3089.3	2856.9	Sym $\nu_{C\text{-}H(Aliphatic)}$
3021.0	2856.9	Sym $\nu_{C\text{-}H(Aliphatic)}$
1670.7	1636.1	$\nu_{C=C}+\nu_{C=N}$
$1636.6_{(Sh)}$	1636.1	$\nu_{C=C}+\nu_{C=N}$
1558.1	1597.1	$\nu_{C=C}+\nu_{C=N}$
1489.1	1492.8	$\delta_{C\text{-}H}+\nu_{C=C}+\nu_{C=N}$
1399.3	1443.9	$\delta_{C\text{-}H}$
1337.5	1357.6	$\nu_{C\text{-}C}+\nu_{C\text{-}N}$
1326.7	-	$\delta_{C\text{-}H}$
1247.5	1259.1	$\delta_{C\text{-}H}+\nu_{C\text{-}C}+\nu_{C\text{-}N}$
1161.0	1131.9	$\delta_{C\text{-}H}+\nu_{C\text{-}C}+\nu_{C\text{-}N}$
1064.0	1041.4	Ring breathing
997.1	1008.3	Ring breathing
830.4	827.7	$\delta_{C\text{-}H\ (pyrazole)}$
809.6	795.5	Ring puckering
768.5	755.5	δ_{CNC}

[a] **ν**: stretching and **δ:** Bending

Table S2 Evaluation of Antioxidant Activity using DPPH scavenging assay for ^{Morph}**BPT.**

Sample conc. (μg/mL)	DPPH scavenging %	S.D. (±)
1280	15.38	0.76
640	12.50	0.42
320	7.93	0.19
160	6.09	0.43
80	4.02	0.26
40	2.17	0.19
20	1.09	0.37
10	0.33	0.15
0	0	0

Table S3 Evaluation of Antioxidant Activity using DPPH scavenging assay for [Co(^{Morph}BPT)Cl_2]; **1.**

Sample conc. (µg/mL)	DPPH scavenging %	S.D. (±)
**1280**	**29.74**	**1.28**
**640**	**16.20**	**1.42**
**320**	**12.83**	**0.61**
**160**	**8.37**	**0.59**
**80**	**6.41**	**0.37**
**40**	**4.13**	**0.25**
**20**	**2.61**	**0.13**
**10**	**1.74**	**0.28**
**0**	**0**	**0**

Table S4 Evaluation of Antioxidant Activity using DPPH scavenging assay for ^{Pip}**BPT.**

Sample conc. (µg/mL)	DPPH scavenging %	S.D. (±)
1280	18.91	0.43
640	15.98	0.74
320	11.30	0.62
160	8.48	0.36
80	5.98	0.34
40	2.39	0.23
20	1.41	0.17
10	0.98	0.24
0	0	0

Table S5 Evaluation of Antioxidant Activity using DPPH scavenging assay for **[Co(^{Pip}BPT)Cl_2]; 2**.

Sample conc. (μg/mL)	DPPH scavenging %	S.D. (±)
1280	18.37	0.95
640	13.91	0.63
320	8.92	0.87
160	7.61	0.35
80	4.78	0.44
40	2.50	0.59
20	1.30	0.26
10	0.65	0.13
0	0	0
